# Abdominal Wall Endometriosis Postcesarean Section: Diagnosis, Surgical Management, and Case Study of Extensive Uterine Adhesion

**DOI:** 10.1155/crog/6018368

**Published:** 2026-02-23

**Authors:** Maryam Hashemi, Maryam Dehghan

**Affiliations:** ^1^ Department of Minimally Invasive Surgery Gynecology, School of Medicine, Al-Zahra Hospital, Isfahan University of Medical Sciences, Isfahan, Iran, mui.ac.ir

**Keywords:** abdominal wall, cesarean section, endometriosis, excision

## Abstract

**Introduction:**

Endometriosis involves ectopic endometrial tissue outside the uterus, commonly in the pelvis but sometimes affecting organs like the urinary tract, gastrointestinal system, and respiratory tract. Abdominal wall endometriosis (AWE) is a rare subtype where endometrial tissue infiltrates the abdominal wall, often occurring secondarily in surgical scars, particularly after cesarean sections. Diagnosing AWE is difficult due to varied symptoms and locations, making imaging techniques such as ultrasound and MRI crucial for evaluation. Preoperative assessment is vital to determine the extent of tissue invasion, especially if large muscles, the peritoneum, or bowel are involved, which may require general surgical intervention.

**Case Presentation:**

A 43‐year‐old woman with a history of three cesarean sections presented with menorrhagia, chronic pelvic pain, and a palpable lump above her cesarean scar. Ultrasonography revealed a hypoechoic irregular mass measuring 39 × 34 mm in the linea alba above the cesarean scar, extending into the peritoneal cavity and adherent to the uterine body. Surgical wide excision of the mass was attempted, but due to its extension and severe adhesion to the uterus, complete excision with clear margins was not possible. So, a total hysterectomy with bilateral salpingo‐oophorectomy was performed.

**Discussion:**

AWE mainly results from iatrogenic implantation of endometrial cells, particularly following cesarean sections, though lymphatic spread and metaplasia are also possible causes. It typically presents as a painful abdominal mass with symptoms like localized pain, swelling, bruising, bleeding, intermittent pelvic pain, and reduced fertility. Diagnosis is primarily made via abdominal and transvaginal ultrasound, with MRI used in uncertain cases. Medical treatments such as oral contraceptives, progesterone, danazol, and GnRH agonists offer only partial symptom relief without curing AWE. The definitive treatment is wide local surgical excision with at least 1 cm margins to prevent recurrence or rare malignant transformation. When the fascia and muscle are involved, or defects exceed 50 mm, fascia mobilization and polypropylene mesh placement may be necessary. In malignant or extensive cases, complete hysterectomy with bilateral salpingo‐oophorectomy may be indicated. Preventive surgical measures during cesarean sections include gentle uterine handling, bleeding control, high‐pressure saline irrigation before closure, avoiding dead spaces, use of wound protectors, thorough abdominal wall cleaning, specimen retrieval bags, and employing new needles and sutures to reduce AWE risk.

**Conclusion:**

AWE is a rare condition with unclear causes, increasingly relevant due to rising cesarean and obstetric procedures. Diagnosis relies on clinical assessment, patient history, ultrasound, and MRI. The primary treatment is wide surgical excision, which may be more extensive for large or complex lesions, with careful follow‐up to monitor for recurrence.

## 1. Introduction

Endometriosis is defined as the presence of ectopic endometrial tissue outside the uterine cavity that can respond to hormonal stimulation from the ovaries [1].

In most cases, endometriosis is located within the pelvis. However, ectopic endometrial tissue can also be found outside the pelvis, affecting various organs and occasionally causing cyclical symptoms [2]. Although extrapelvic disease is uncommon, cases involving the urinary tract, gastrointestinal system, and respiratory tract have been previously reported [1].

Abdominal wall endometriosis (AWE), defined as the presence of endometriotic infiltration in any segment or depth of the abdominal wall, is a rare manifestation of endometriosis [3, 4]. Spontaneous AWE occurs in the abdomen without scarring and accounts for approximately 20% of all AWE cases. Secondary AWE typically develops in surgical scars resulting from gynecological or obstetric procedures, with the highest incidence following cesarean sections (57%–92%) [4, 5]. The reported incidence of AWE ranges from 0.03% to 3.5%, while the overall incidence of AWE within cesarean section scars is reported to be between 0.03% and 0.45% [4, 6].

Given the difficulty in diagnosing AWE due to its wide range of clinical symptoms, variable location of the abdominal mass, and numerous possible differential diagnoses, ultrasound and magnetic resonance imaging (MRI) of the pelvis and abdomen—including evaluation of the abdominal wall—play a crucial role in diagnosis [3, 7].

It is important to remember that preoperative assessment of invasion is crucial. In cases where there is invasion of large muscles or deep invasion of the peritoneum or bowel, the involvement of a general surgeon is essential, along with all its logistical implications [8].

Herein, we present a case of AWE extending beyond the peritoneum and adhering to the uterine wall following multiple previous cesarean sections.

## 2. Case Presentation

A 43‐year‐old woman with a history of three cesarean sections presented with symptoms of menorrhagia and chronic pelvic pain. Palpation of the abdomen revealed a lump above the cesarean scar. Ultrasonography showed mild adenomyosis, decreased sliding in the pouch of Douglas, and a hypoechoic, irregular mass measuring approximately 39 × 34 mm in the linea alba, located above the cesarean scar, 8 mm from the skin surface. The mass extended into the peritoneal cavity and appeared to be adherent to the uterine body. Both ovaries had a normal appearance. Hormonal tests related to abnormal vaginal bleeding, including thyroid function tests, prolactin, and beta‐hCG, showed no pathological findings. The pathology report from the pipelle biopsy performed in the clinic prior to surgery indicated proliferative endometrium consistent with the menstrual cycle.

She then underwent surgical wide excision of the mass. The surgery was conducted in the operating room of a Level III hospital, which has access to a multidisciplinary team comprising a skilled general surgeon, laparoscopic surgeon, vascular surgeon, and urologist, due to the anticipated need for collaborative expertise.

The mass was extended into the peritoneal cavity and adhered to the uterine body, so it could not be excised with grossly free margins. Due to the severe adhesion of the mass to the uterine corpus, the occurrence of uterine adenomyosis, and the presence of superficial endometriosis lesions on the ovaries, a decision was made to perform a total hysterectomy and bilateral salpingo‐oophorectomy (Figure 1). The patient passed the postsurgical period uneventfully and was discharged in good condition.

Figure 1(a) Abdominal wall endometriosis is adherent to the uterine body. (b) Uterus and adnexa after removal.

**Figure:**
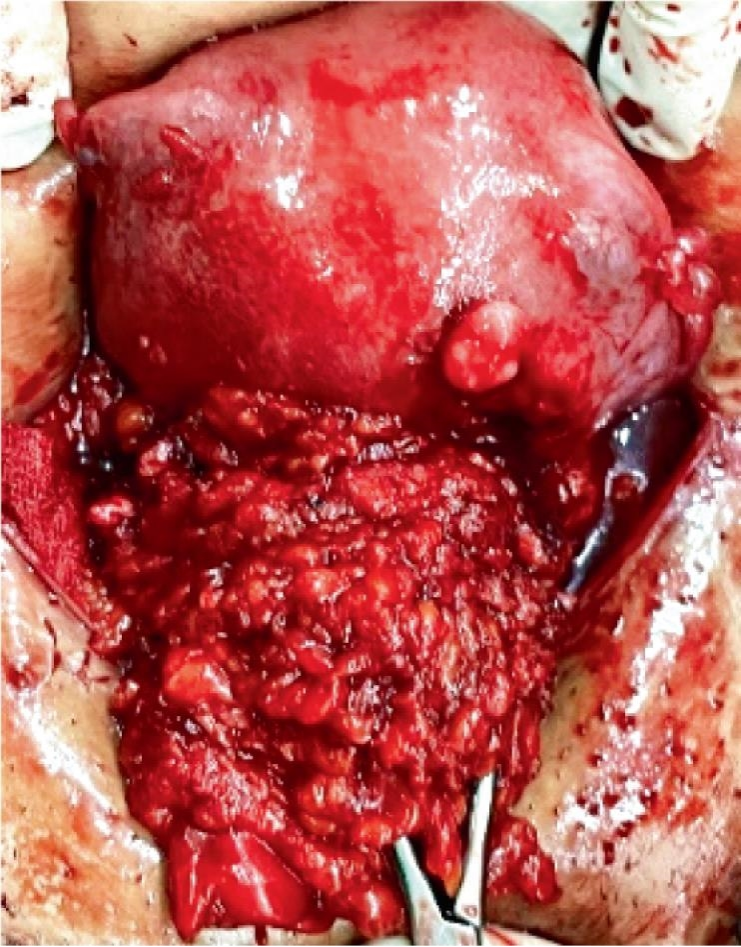
(a)

**Figure:**
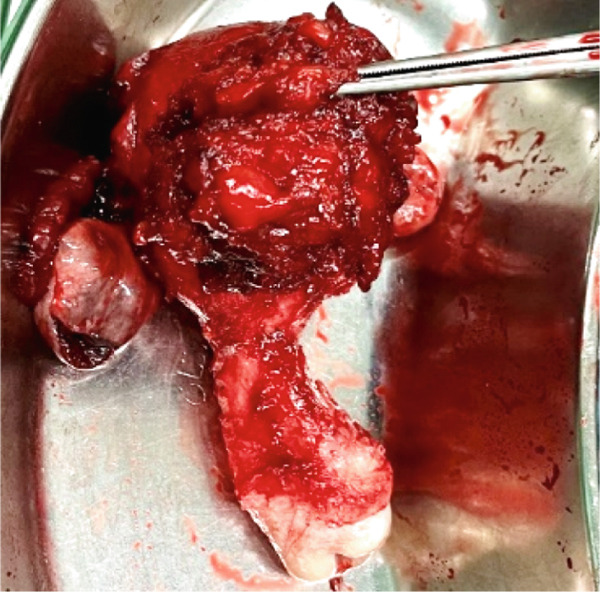
(b)

The histopathological evaluation of the specimen revealed AWE, uterine adenomyosis, and bilateral ovarian endometriosis.

## 3. Discussion

Although the exact pathogenesis of AWE remains unclear, several theories have been proposed [3]. The most widely accepted hypothesis is that endometrial cells are directly implanted through an iatrogenic process. Other proposed mechanisms include lymphatic or hematogenous dissemination, metaplastic transformation, and cellular immune modification [9]. Given that our patient′s mass extended entirely from the previous cesarean section site to the abdominal wall, it is most likely that an iatrogenic process resulting from prior cesarean sections is responsible.

The clinical presentation of AWE varies. Some women experience severe pain, while others may be asymptomatic. Common complaints include a painful mass, localized discomfort, swelling, bruising, or bleeding in the affected area. Intermittent pelvic pain and reduced fertility may also occur. Our patient′s primary complaint was chronic pelvic pain, particularly during menstruation, which appeared to result from the mass extending into the pelvic cavity and its association with adenomyosis [4].

In addition to clinical signs and symptoms, ultrasound imaging of the abdomen, including the abdominal wall, can help determine the extent of endometriotic implants and is considered the first‐line imaging modality in the preoperative evaluation of AWE [4, 10]. If ultrasound findings are inconclusive, an MRI is recommended. In our patient, a combination of abdominal and transvaginal ultrasound provided valuable and accurate information about the uterus, adnexa, and the extent of the mass, eliminating the need for MRI.

Although drug therapies such as oral contraceptive pills, progesterone, danazol, and GnRH agonists can be useful in treating endometriosis, these medications are not effective in curing patients with AWE and provide only partial symptom relief [1, 2]. The preferred treatment for AWE is surgical excision with wide local margins of at least 1 cm to prevent recurrence or malignant transformation. Although malignant transformation of endometriosis is rare, with a reported incidence of 1%, it should not be overlooked [3]. In cases where the underlying abdominal wall fascia and muscle are affected, when there may be tension at the suture line, or in cases where the defect is larger than 50 mm, extensive mobilization of the fascia and placement of a polypropylene mesh may be recommended [4].

The gold standard treatment for AWE is wide local excision with clear margins, but cases of complete hysterectomy with bilateral salpingo‐oophorectomy have been reported. These are often seen in cases of malignant transformation, particularly to clear cell or endometrioid carcinoma, sometimes with extension to the pelvic cavity [11–13]. The decision to perform a total hysterectomy with bilateral salpingo‐oophorectomy was based on several factors: the patient had completed her family, the presence of uterine adenomyosis, and her complaint of menorrhagia that was resistant to medical treatment. Additionally, the mass was adherent to the uterus, making it impossible to separate the mass with clear margins without damaging the uterus. Furthermore, superficial endometriosis lesions were present on the surface of the ovaries and peritoneum, increasing the likelihood of recurrence if definitive treatment was not provided.

Various methods have been described in gynecological surgery to prevent AWE. It is recommended to gently palpate the uterine tissue, carefully control bleeding, irrigate the intra‐abdominal cavity with high‐pressure saline solution before wound closure, and prevent the formation of subcutaneous dead spaces [2, 14]. A recent report of 83 cases of AWE recommends the use of wound protectors or retractors in all cesarean sections; if this is not possible, vigorous irrigation and thorough cleaning of the abdominal wall with saline solution should be performed. Additionally, the use of an endoscopic specimen retrieval bag (Endo Bag) to remove surgical specimens is advised. The report also emphasizes using new needles and sutures to close the abdominal wall layers in procedures requiring suturing of both the uterus and the abdominal wall [4].

## 4. Conclusion

AWE is a rare clinical condition with an unclear pathophysiology. Although uncommon, obstetricians and gynecologists should be familiar with this condition due to the increasing rates of cesarean sections and other obstetric procedures. Diagnosis is based on clinical evaluation, patient history, ultrasound, and MRI. Surgical wide excision remains the primary treatment and may require more extensive surgery in cases of large or complex lesions. Follow‐up is essential because of the risk of recurrence. Although rare, AWE can extend into the abdominopelvic cavity and adhere to the uterus. Therefore, it is recommended to obtain informed consent for total abdominal hysterectomy and bilateral salpingo‐oophorectomy if these procedures become necessary during surgery.

## Author Contributions

M.D. and M.H. participated in the patient management and writing of this manuscript.

## Funding

No funding was received for this manuscript.

## Ethics Statement

This article does not contain any studies involving human participants performed by any of the authors.

## Consent

Written informed consent was obtained from the patient for publication of this case report and any accompanying images.

## Conflicts of Interest

The authors declare no conflicts of interest.

## Data Availability

The data that support the findings of this study are available from the corresponding author upon reasonable request.
